# Glycoprotein Quality Control and Endoplasmic Reticulum Stress

**DOI:** 10.3390/molecules200813689

**Published:** 2015-07-28

**Authors:** Qian Wang, Jody Groenendyk, Marek Michalak

**Affiliations:** Department of Biochemistry, University of Alberta, Edmonton, AB T6G 2H7, Canada; E-Mails: qw7@ualberta.ca (Q.W.); jlg2@ualberta.ca (J.G.)

**Keywords:** endoplasmic reticulum, calnexin, calreticulin, chaperone, stress

## Abstract

The endoplasmic reticulum (ER) supports many cellular processes and performs diverse functions, including protein synthesis, translocation across the membrane, integration into the membrane, folding, and posttranslational modifications including *N*-linked glycosylation; and regulation of Ca^2+^ homeostasis. In mammalian systems, the majority of proteins synthesized by the rough ER have *N*-linked glycans critical for protein maturation. The *N*-linked glycan is used as a quality control signal in the secretory protein pathway. A series of chaperones, folding enzymes, glucosidases, and carbohydrate transferases support glycoprotein synthesis and processing. Perturbation of ER-associated functions such as disturbed ER glycoprotein quality control, protein glycosylation and protein folding results in activation of an ER stress coping response. Collectively this ER stress coping response is termed the unfolded protein response (UPR), and occurs through the activation of complex cytoplasmic and nuclear signaling pathways. Cellular and ER homeostasis depends on balanced activity of the ER protein folding, quality control, and degradation pathways; as well as management of the ER stress coping response.

## 1. Introduction

The endoplasmic reticulum (ER) is a multifunctional network of intracellular membranes responsible for the secretory protein demands of the cell as well as adaptive responses to stress. Proteins within the ER are responsible for controlling the translation, folding, and translocation of nascent polypeptides for secretion or insertion into the membrane as transmembrane proteins. The many functions of the ER are supported by its luminal environment, including formation of disulphide bonds and protein folding, carried out by molecular chaperones and folding enzymes, as well as post-translational modifications [1]. Once soluble proteins are properly folded, they are targeted to the secretory pathway. The molecular chaperones of the ER are also important for regulating intracellular Ca^2+^ signaling within the ER lumen and the rest of the cell [2,3,4]. Accumulation of mis-folded proteins in the ER due to cellular insults, impaired ER homeostasis and/or disrupted glycoprotein quality control leads to activation of a specific ER stress coping response, termed the unfolded protein response (UPR) [5,6]. The UPR results in ER to nucleus and ER to plasma membrane signaling, with activation of genes encoding ER chaperone expression [6], inhibition of protein synthesis and increased protein degradation [5,6]. Consequently, the ER may be defined as a versatile component of the intracellular reticular network able to detect and integrate incoming signals, modulate and respond to its own luminal dynamics and generate output signals in response to environmental changes [4,5,7].

## 2. Glycoproteins and ER Quality Control

**Figure 1:**
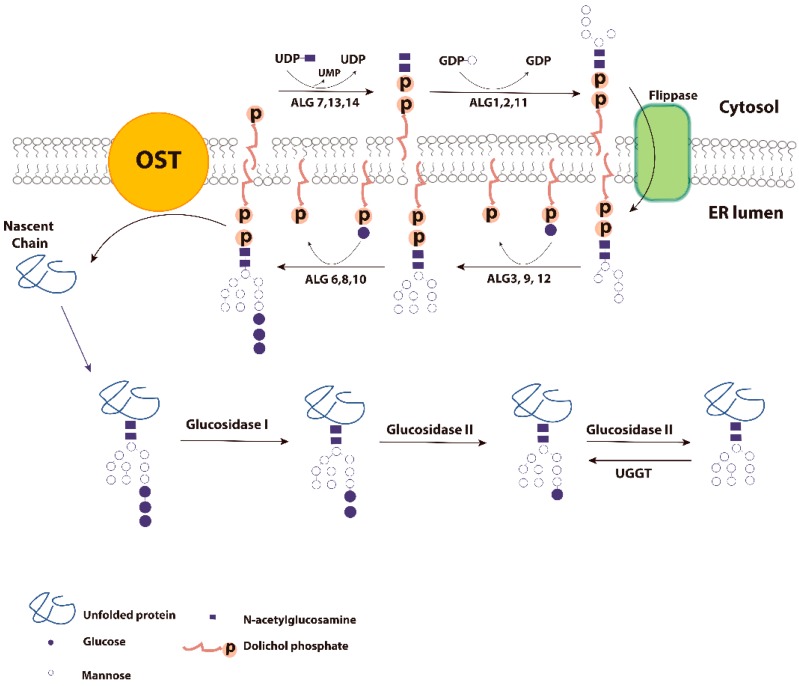
*N*-Glycan synthesis and glucose trimming in the ER. The synthesis of *N*-glycans begins on the cytoplasmic face of the ER membrane. The enzymes that catalyze each step in *N*-glycan biosynthesis are encoded by ALG genes. Firstly, GlcNAc-P is attached to the membrane-bound dolichol phosphate from UDP-GlcNAc by GlcNAc-1-phosphotransferase (ALG 7) and UMP is released. The addition of GlcNAc and mannose residues is catalyzed by ALG13/14, and ALG 1, 2, and 11 sequentially. The partially synthesized *N*-glycan (GlcNAc_2_Man_5_) is flipped across the ER membrane to the luminal side by an ATP-independent flippase. Four mannose and three glucose residues are added to generate the final mature *N*-glycan which is transferred to the nascent polypeptide chain by oligosaccharyltransferase (OST). The terminal two glucoses can be removed by glucosidase I and glucosidase II separately. Glucosidase II also removes the last glucose, which can be re-attached by UGGT.

Secretory (glyco)proteins and membrane (glyco)proteins are synthesized on ER membrane-bound ribosomes. Most of the nascent protein chains that enter the ER lumen are targeted for *N*-linked glycosylation (Figure 1). The covalent attachment of hydrophilic oligosaccharide to the nascent chain can increase protein solubility and stability. Initially, the *N*-glycan is partially synthesized on the cytoplasmic side of the ER membrane. The *N*-glycan is then “flipped” to the ER luminal side by a bi-directional flippase in an ATP-independent manner [8]. Future addition of mannose and glucose moieties on the partially synthesized *N*-glycan is carried out by multiple mannosyltransferases and glycosyltransferases to form the mature oligosaccharide donor for protein *N*-glycosylation [9]. A specific 14 residue oligosaccharide consisting of Glc_3_Man_9_GlcNAc_2_ (Glc: Glucose; Man: Mannose; GlcNAc: *N*-acetylglucosamine) is transferred from the donor Glc_3_Man_9_GlcNAc_2_-PP-dolichol to the Asn-X-Ser/Thr (NXS/T, where X is any amino acid except proline, although in some case NXC, NXV or NG can also be used [10]) site in the growing polypeptide chain, occurring when the polypeptide termini is a minimum of 12–14 amino acids away from the membrane [11]. This transfer is catalyzed by OST (oligosaccharyltransferase), an ER membrane bound and translocon (Sec61) associated multimeric protein complex [12,13,14]. Immediately after the attachment of Glc_3_Man_9_GlcNAc_2_ to the nascent chain, the terminal glucose residue is trimmed by glucosidase I. Since both OST and glucosidase I are part of the translocon complex [12], they can closely associate with newly synthesized polypeptide chain, with the oligosaccharide transferring and the first glucose trimming generally occurring co-translationally [15,16,17,18]. Glucosidase I is an 85 kDa type II membrane glycoprotein composed of two contiguous domains: a membrane bound domain and a 39 kDa catalytic luminal domain [18]. This first glucose trimming process is very fast and occurs immediately after the glycan attaches to the nascent chain [15,16]. The Glc_2_-*N*-glycan generated by glucosidase I can associate with malectin, an ER localized type 1 membrane associated *N*-glycan binding protein [19]. The possible functions of malectin include recruiting glucosidase II for further deglycosylation and preventing aggregation of nascent polypeptides during the early synthesis period [19].

The next two glucose residues are sequentially removed by glucosidase II [20]. When glucosidase II removes the second glucose from the core oligosaccharide, it generates the monoglucosylated glycan (GlcMan_9_GlcNAc_2_) that allows the initial binding of the *N*-glycosylated nascent chain to calnexin and calreticulin, members of the protein quality control cycle [21,22]. Glucosidase II is a soluble heterodimeric enzyme that removes the remaining two glucose residues from *N*-glycan [20] and is composed of a catalytic α subunit and a regulatory β subunit [20]. The regulatory β subunit contains a mannose 6-phosphate receptor homology (MRH) domain and KDEL ER retrieval signal [23,24]. *In vitro* affinity chromatography shows that the glucosidase II β subunit can bind strongly to glycans with the α 1,2-linked mannobiose structure. Moreover, mutations in the β subunit can significantly inhibit glucosidase II substrate binding [24]. 

**Figure 2:**
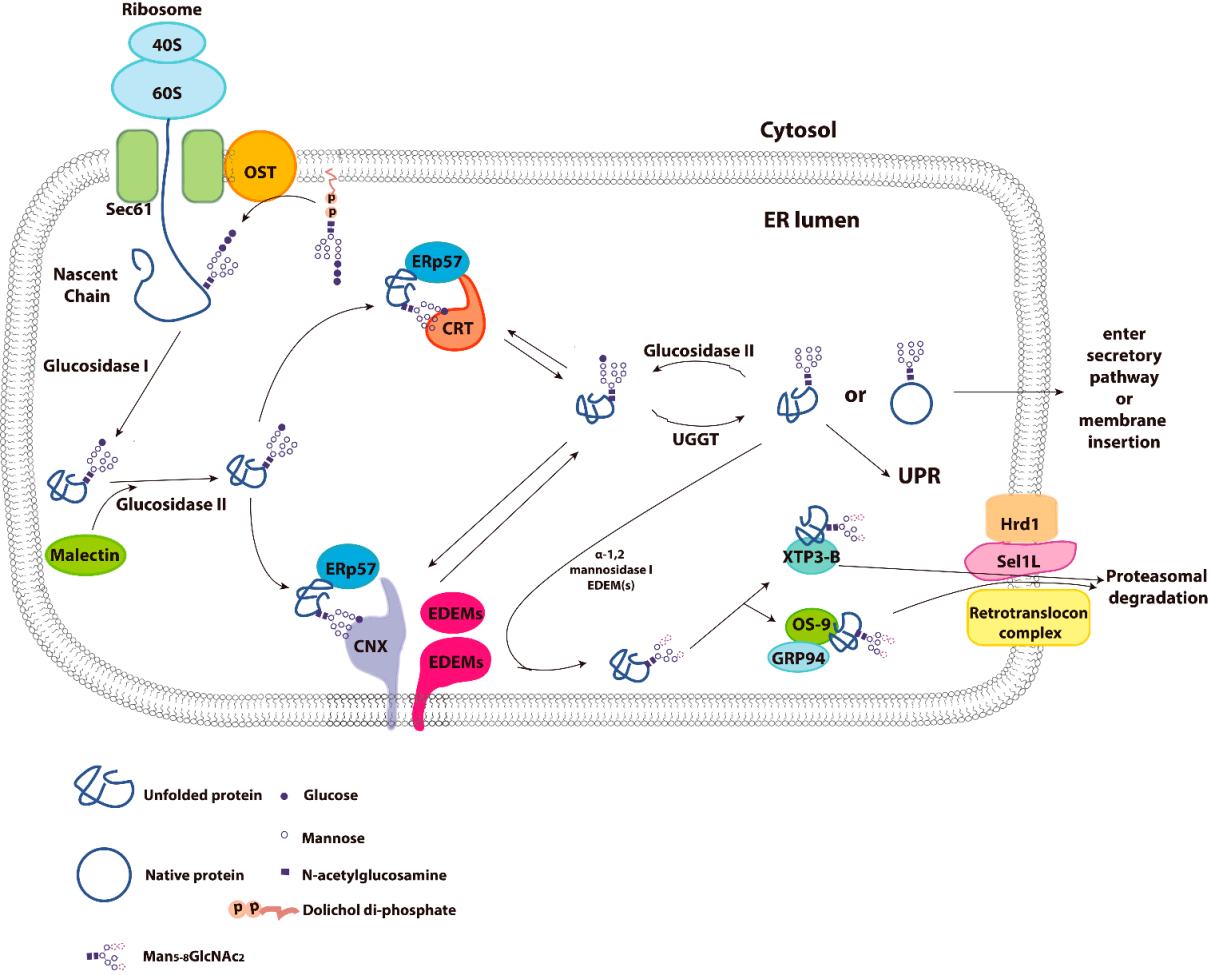
Glycoprotein folding in ER and the calnexin/calreticulin cycle. The folding of newly synthesized glycoproteins in the ER is assisted by calnexin (CNX) and calreticulin (CRT). Initially, a 14-residue oligosaccharide is transferred from dolichol di-phosphate to the nascent chain of glycoprotein by oligosaccharyltransferase (OST). The terminal two glucoses are removed by glycosidase I and II sequentially. The monoglucosylated form of the glycoprotein can then bind to CNX and the CRT N-domain to facilitate its folding. Both CNX and CRT are associated with ERp57 on their arm-like P-domains. The binding of CNX and CRT with the protein substrate is also stabilized by the oxidoreductase ERp57. Removal of the last glucose by glucosidase II causes substrate release from CNX/CRT. If the protein reached its native conformation, it will be released from the ER through the protein secretory pathway. However, if the protein is not correctly folded, the exposed hydrophobic region will be recognized by UDP-glucose:glycoprotein glycosyltransferase (UGGT). This enzyme will add the removed glucose back on to the glycoprotein from a UDP-glucose donor. This generates a monoglucosylated *N*-glycan on the glycoprotein and promotes binding with CNX/CRT again. A protein may cycle through this CNX/CRT cycle numerous times before it reaches its native conformation. However, if proper folding of the protein still cannot be achieved, the unfolded protein will be targeted to the ER associated degradation (ERAD) pathway. ERAD targeted proteins undergo sequential demannosylation assisted by EDEM, an ER-resident mannosidase. Demannosylated ERAD substrates are then recognized by and bind to XTP3-B and OS-9 which prevent aggregation and are then further targeted to Hrd1-SEL1L for ubiquitination. This is followed by retro-translocation into the cytoplasm where the misfolded polypeptide chain is subjected to proteasome degradation. Additionally, the accumulation of unfolded protein will also trigger the unfolded protein response (UPR).

The NMR structure of this MRH domain demonstrated a β-barrel fold similar to the mannose binding site of other mannose 6-phosphate receptors [25]. However, this mannose binding site on the glucosidase II β subunit is very shallow, and can only fit a single mannose residue [25]. Unlike glucosidase I, the trimming of glucose by glucosidase II is more regulated. The second glucose trimming by glucosidase II only occurs when there is a second glycan present in the nascent chain [15]. After removal of the second glucose by glucosidase II, the resulting monoglucosylated glycoprotein can bind to the lectin chaperones, calnexin and calreticulin, and the protein is targeted to the ER protein quality control cycle (Figure 2).

## 3. Calnexin/Calreticulin Cycle 

Calnexin and calreticulin are two critical ER localized lectins, non-classical chaperones, responsible for the folding and quality control of newly synthesized glycoproteins (Figure 2) [1]. Calnexin is a Type I integral membrane protein, with the bulk of the polypeptide exposed to the lumen of ER where the protein interacts with substrates. The cytoplasmic tail of calnexin may also affect protein folding and appears to interact with a number of cytoplasmic molecules, as well as undergoing posttranslational modification including phosphorylation, sumoylation and palmitoylation [26,27,28,29,30,31]. Calreticulin is a high capacity Ca^2+^ buffering ER localized protein, with similar substrate specificity for glycoproteins as calnexin [32]. Both protein, in conjunction with ERp57, an ER resident oxidoreductase, function as the major chaperone complex in the calnexin/calreticulin cycle. Calnexin or calreticulin interact with the monoglucosylated glycan found on the nascent polypeptide chains. The sugar residues on the glycoprotein interact with the globular N-domain of calnexin or calreticulin, while the polypeptide chain can form transient mixed disulfide bonds with ERp57 to further stabilize the interaction and assist in folding [1]. The binding of these non-stable glycoprotein intermediates with calnexin/calreticulin-ERp57 complexes can also prevent protein aggregation. When the third glucose residue is removed by glucosidase II, allowing its release from the calnexin and calreticulin protein quality control cycle [1,33], then the native glycoprotein is released from the ER and transits through the secretory pathway. Since glucosidase II seems to have no substrate specificity towards native glycoproteins, it can also cleave the third glucose from improperly folded proteins. The last glucose of the *N*-linked glycan is removed by glucosidase II upon substrate release from calnexin/calreticulin, but can be re-attached by the UDP-glucose:glycoprotein glucosyltransferase (UGGT) [34]. UGGT is a fascinating glucosyltransferase that plays an essential role in ER protein quality control. It can re-attach the third glucose (previously removed) onto the *N*-linked glycan of improperly folded glycoproteins, which allows the glycoprotein to re-enter the calnexin/calreticulin protein quality control cycle [35]. UGGT is an enzyme comprised of two main functional parts: a large N-terminal folding sensor region, and a C-terminal carbohydrate transferase domain [1,36]. The N-terminal sensor region is predicted to contain three tandem thioredoxin-like domains [36]. *In vitro* molecular studies demonstrate that UGGT only interacts with the hydrophobic amino acids exposed in denatured protein conformations [37]. Until recently, the crystal structure of the third thioredoxin-like domain in the N-terminal sensor region had not been solved. The crystal structure revealed an extensive hydrophobic patch, which is concealed by a single α helix [36]. Functionally, this indicates that hydrophobic amino acids of other proteins can bind to UGGT through hydrophobic interactions [36]. This may explain the substrate selectivity of UGGT towards misfolded proteins, as the hydrophobic amino acid side chains in mis-folded proteins are more likely to be exposed than in properly folded proteins. A protein may enter this quality control cycle numerous times until it is properly folded. However, if correct folding of the protein cannot be achieved, the mis-folded protein will be targeted for degradation through the ER associated degradation (ERAD) process. Accumulation of misfolded protein will also trigger the unfolded protein response (UPR). These mechanisms function to ensure that misfolded and incorrectly assembled proteins are retained in the ER and eventually degraded. Other than calnexin and calreticulin, there are additional ER chaperones involved in protein folding, including BiP (immunoglobulin binding protein) and PDI (protein disulfide isomerase) [1,38,39].

## 4. Molecular Properties of Calnexin and Calreticulin

Calnexin is a 90-kDa, non-glycosylated type I membrane protein [40,41]. The crystal structure of calnexin’s ER luminal domain was solved at 2.9Å resolution and revealed two important functional domains in its core luminal region, namely the P-domain and the globular carbohydrate binding domain [41,42]. The C-terminus acidic cytoplasmic tail of calnexin was not included in crystallization studies [42]. The P domain consists of a long extended arm (134 amino acids in length) composed of β-sheets and loops that are rich in proline residues. NMR analysis shows that the P domain of calnexin contains the ERp57-binding site on the tip of the P domain at residues 361-367 [43]. This interaction with ERp57 is important for the chaperone function of calnexin. A β-sandwich structure forms the carbohydrate binding domain of calnexin, which is commonly found in leguminous lectins [42].

Calreticulin is a 46-kDa ER luminal protein [32]. Recent evidence points to the targeting of calreticulin to alternate sites within the cell, including the nucleus, mitochondria, cytoplasm and the plasma membrane [44,45,46,47,48,49]. Additional studies are needed to further support the diverse intracellular localization of this ER resident protein and it is outside the scope of this review. While the crystal structure of the whole protein has not been solved, calreticulin shows a high degree of primary amino acid sequence similarity to calnexin. It contains three structural and functional domains: a globular N-domain, a long P-domain arm, and an acidic C-domain [32]. The crystal structure of the globular N-domain and NMR analysis of calreticulin shows that calreticulin bears strong homology to calnexin. Moreover, the ERp57 binding site also lies at the tip of the P-domain, specifically at polypeptide segment 225-251 [50]. The primary oligosaccharide binding site in calreticulin is located in the globular N-domain [51]. There is potentially a secondary binding site in the P-domain, but it shows much weaker interactions with sugar and a lack of specificity for monoglycosylated oligosaccharides [51]. Mutational analysis studies identified that mutation of some residues in the N-domain, including Tyr109, Asp135 [52], Tyr128, Tyr109, Lys111, and Asp317 [53], can completely abolish oligosaccharide binding. A high resolution crystal structure of the N-domain in complex with its tetrasaccharide substrate (GlcMan_9_GlcNAc_2_) suggests that the shape of the sugar binding pocket is formed by concave β-sheets with two residues, Gly124 and Lys111, responsible for the binding selectivity and specificity of monoglycosylated oligosaccharides to calreticulin. These two residues can form direct hydrogen bonds with the oxygen of the glucose [54].

## 5. Endoplasmic Reticulum Associated Degradation (ERAD)

ERAD is a process by which misfolded ER proteins are detected in the protein secretory pathway by ER-resident factors and directed to translocation machinery for retro-translocation into the cytoplasm, where they undergo ubiquitin- and proteasome-dependent degradation. ERAD is initiated when the misfolded proteins are recognized by EDEM (ER degradation-enhancing α-mannosidase–like protein), which starts to trim the mannose residues from the core glycan of misfolded proteins [55,56,57,58]. Recent evidence points to compartmentalization as the mechanism to target proteins for ERAD [59,60]. Mannose removal involves several proteins, including ERManI (ER α1,2-mannosidase I), EDEM1,2,3 and Golgi resident mannosidase I [56,61,62]. Interestingly, ERManI is localized in quality control vesicles [61,63], while EDEM1 is found mostly in autophagy like vesicles that do not involve the COPII exit sites [56,64,65]. Finally, the Golgi-resident α1,2-mannosidases [62] cleave mannose residues from Man_9_GlcNAc_2_ to Man_5_GlcNAc_2_ of the *N*-glycan [9,66]. Mannose trimming is believed to be the key step for preventing ERAD targeted proteins to re-enter the calnexin/calreticulin cycle [67,68]. Decreased mannose content in the core glycan of glycoproteins can prevent glycosylation by UGGT in cell-free assays [69], as well as binding to calreticulin and calnexin [22]. However, *in vivo*, UGGT enzymatic activity does not influence the mannose content in the core glycan of the substrate [70]. The exit of misfolded glycoproteins from the calnexin/calreticulin cycle most likely occurs upon removal of the outermost mannose residue from the glucose-containing arm, preventing re-glycosylated by UGGT [66]. The deglycosylated and demannosylated misfolded proteins are recognized by the ERAD lectin, osteosarcoma amplified 9 (OS-9) and XTP3 transactivated protein or erlectin (XTP3-B), which bind to the misfolded proteins and delivers them to HRD1-SEL1L for ubiquitination [71,72]. The HRD1-SEL1L ubiquitin ligase is part of a complex in the ER membrane which includes Der1-like proteins 1 and 2, VCP, p97, valosin containing protein (VCP)/p97-interacting membrane protein (VIMP), and Herp [73,74]. This is followed by retro-translocation into the cytoplasm and subsequently targeting to the 26S proteasome for degradation [75]. The retro-translocation machinery may include Derlin-1 [76], which contains four transmembrane regions, forming a complex with the small membrane protein VIMP, cytoplasmic valosin containing protein (VCP) also termed AAA ATPase p97 [73,77,78], and N-glycanase, which removes the oligosaccharide [79]. This complex appears to be distinct from the Sec61 translocon [73,80,81]. 

## 6. Quality Control and Endoplasmic Reticulum Stress

Protein glycosylation is a fundamental part of the ER protein quality control. Entry to the secretory pathway begins at the ER to drive glycoprotein movement towards the trans-Golgi to deliver properly folded glycoproteins. Many of the cell surface glycoproteins are critical for the homeostasis of eukaryotic cells. Impaired protein glycosylation and folding may trigger activation of ER stress coping responses (*i.e.*, the UPR). Furthermore, disruption of glycoprotein trafficking to the cell surface may also contribute to the activation of ER stress coping responses and the cell’s ability to recognize and deal with environmental stimuli.

One of the main features of ER stress is an accumulation of mis-folded proteins in the ER lumen, which results in activation of the UPR [82]. The UPR triggers an adaptive response to restore ER homeostasis by coordinating a reduction in the quantity of protein expressed, increased expression of molecular chaperones to deal with buildup of misfolded protein, as well as an increase in ER-associated protein degradation to remove misfolded proteins [83,84]. Initially, suppression of protein synthesis and up regulation of ER chaperones such as calreticulin and BiP attempt to deal with the accumulation of mis-folded protein, but if the condition continues or becomes more severe, the UPR will trigger apoptosis as a means to eliminate the problem [85]. To maintain homeostasis of ER protein folding, several ER transmembrane proteins are involved: activating transcription factor 6 (ATF6), protein kinase RNA-like ER kinase (PERK) and inositol-requiring protein 1α (IRE1α), with the luminal domains of these proteins serving as ER stress-sensing domains via an interaction with BiP [86,87]. BiP interacts with the luminal domain of these transmembrane proteins under non-stressed conditions. With accumulation of unfolded proteins, BiP binds to the unfolded hydrophobic portions of the misfolded protein. This activates the intrinsic protein kinase activity of IRE1α, which controls the endoribonuclease activity-dependent cleavage of XBP1 mRNA, a transcription factor involved in feedback regulation of protein expression and degradation [88]. Another member of the UPR, PERK is an ER kinase that phosphorylates eukaryotic translation initiation factor 2α (eIF2α) and attenuates protein translation [89] while the third member, ATF6, is released from the ER and is cleaved by Golgi enzymes, site 1 and site 2 proteases (S1P and S2P), to generate a cytoplasmic transcription factor that activates expression of chaperones involved in protein folding and degradation [90]. Specifically, UPR signaling induces several ER chaperone and degradation proteins to decrease the amount of misfolded protein in the stressed cell [87]. These are all mechanisms that a cell employs to minimize damage, recover homeostasis and avert apoptosis.

As an example of the necessity of ER homeostasis, targeted disruption of ER specific proteins can result in embryonic lethality in mice. Disruption of calreticulin, a Ca^2+^ buffering chaperone of the ER, is embryonic lethal at day 13.5 due to malformation of the heart [91]. BiP is embryonic lethal in mice at very early stages of development, embryonic day 3, and is necessary during hatching and implantation [92]. GRP94-deficient mice are embryonic lethal at day 7 due to impaired cardiogenesis [93]. Deletion of the oxidoreductase ERp57 is embryonic lethal at very early stages [94]. Interestingly, calnexin deficiency in mice is not lethal but leads to neurological and metabolic disorders [95].

Fluctuations in ER homeostasis may result in temporary activation of the UPR with translation attenuation and an increase in the level of protein folding chaperones. But upon extended or severe ER stress, the cell will trigger apoptosis. A number of disease states have demonstrated the integral involvement of ER stress, including metabolic disease, cardiovascular disease, neurodegenerative disease and cancer. Metabolic diseases, such as diabetes, have recently been linked to ER stress with an up-regulation of ER stress-associated genes observed in diabetic individuals [85]. Furthermore, targeted disruption of the *chop* gene impedes ER stress mediated diabetes in Akita mice [85], implying that apoptosis is part of the stress response. Reports also establish a role for ER stress in the heart, with ischemia activating the ATF6 dependent branch of the ER stress response [96,97], with sustained stress such as pressure overload causing cardiac expression of ER chaperones [98] or during severe stress such as ischemia/reperfusion with overexpression of ATF6 reducing the amount of necrosis and apoptosis [99]. Similarly, involvement of the ER in neurodegenerative diseases such as Parkinson’s and prion-related disorders has now been documented. Disruption in the secretory pathway appears to be an initiating factor in the proteostasis dysfunction of Parkinson’s disease [100]. Interestingly, ER stress appears to play a dual role by preserving cell survival during early stages of the disease and triggering neuronal degeneration when stress levels are persistent [101]. In Alzheimer’s and prion disorders, UPR activation is seen in post mortem brain samples as well as in mouse models of neurodegeneration [102,103]. ER stress may also contribute to cancer conditions, with recent work showing that three independent mutations in IRE1 are linked to glioblastoma or hepatocellular carcinoma [104,105,106]. As well, XBP1 splicing is increased in a triple negative breast cancer, leading to an aggressive phenotype [107].

During ER stress, adaptive mechanisms are in place to modify cellular pathways to compensate for the disruption in ER homeostasis. However, some of these modifications can be detrimental to the organism. These adaptive mechanisms lead to alterations in the level of proteins, thereby modifying ER homeostasis [6,108]. These changes include increased expression of extracellular matrix proteins, causing cellular fibrosis [109], variation in Ca^2+^ homeostasis due to expression of Ca^2+^ buffering proteins in the ER [110], and changes in the secretion of proteins that transit through the ER that have downstream effects, such as insulin or other growth factors [111]. A number of signaling proteins also have an ER stress element or an UPR element in their promoter [112], so the ER stress pathway has far reaching effects, both immediate and long term.

## 7. Conclusions

The ER performs many varied functions within the cell, including Ca^2+^ storage, protein folding, quality control and post translational modification; as well as managing stress. Connection between the Ca^2+^ and protein quality control system with the ER stress coping responses such as the UPR is necessary for normal function of the cell, and is required for proper cell differentiation and growth, tissue biogenesis and organism embryogenesis. With the multiple functions of the ER, disruption in protein folding and glycoprotein processing results not only in organelle disease but also has detrimental effects at the cellular and systemic levels. Consequently, the ER quality control and stress responses are essential for the growth and subsequent well-being of the organism.
